# Administration of Anesthesia in a Patient with Allgrove Syndrome

**DOI:** 10.1155/2012/109346

**Published:** 2012-04-05

**Authors:** Ayse B. Ozer, Omer L. Erhan, Cevdet Sumer, Ozden Yildizhan

**Affiliations:** Anaesthesiology and Reanimation Department, Faculty of Medicine, Firat University, 23119 Elazig, Turkey

## Abstract

The aim of the present paper is to report the anesthesia administration to a patient who was planned to undergo Heller myotomy for achalasia. There wasnot property in the patient whom allgrove syndrome was excepted any steroid treatment in preoperative period. The night before the operation 18 mg of prednisolone was administered intravenously. Induction of anesthesia was performed with thiopental sodium, vecuronium and fentanyl and the patient received endotracheal intubation. Eyes were taped closed and protected with ointment during surgery. Maintenance of anesthesia was achieved with 2% sevoflurane concentration in 50% O_2_-50% N_2_O. 25 mg of prednisolone was infused preoperatively, and intervention with insulin treatment was initiated when blood glucose level rose to 18 mmol/L at 2 hours. Safe anesthesia can be achieved by observing the preoperative development of tracheal aspiration, adrenal insufficiency and, autonomic dysfunction carefully and maintaining eye protection.

## 1. Introduction

Allgrove syndrome (AS), which isolates adrenal insufficiency, achalasia of the esophageal cardia, and reduction in eye-tear production, and deficient tear production, was first described by Allgrove and his colleagues in 1978 [1]. The disease, also known as triple A or 4A syndrome, is an autosomal recessive disorder associated with autonomic dysfunction, adrenal insufficiency due to ACHT, alacrima, and achalasia [2]. The disease has been supposed to be in relation to ALADIN and AAAS genes, in the vicinity of the type 2 keratin gene clusters, during localization of 12q13 chromosome [3, 4].

Allgrove syndrome generally initiates with the classic findings of primary adrenal insufficiency such as hypoglycemic seizures and shock. Some nonspecific symptoms such as muscle weakness, dizziness and, slow weight loss may also accompany the clinical manifestation [5, 6]. Patients are admitted to hospital with complaints of recurrent vomiting, dysphagia, and growth retardation due to achalasia, besides crying without tears, keratoconjunctivitis sicca, optic atrophy and pupil anomalies associated with alacrima [7–9]. Neurological influences are in relation to peripheral, central and autonomic nervous systems. Microcephaly, mental retardation and learning difficulties may be observed during early life stages. Patients may display progression to bulbospinal amyotrophy, dysarthria, ataxia, muscle weakness, myoclonus, hyperreflexia, and polyneuropathy in the course of time. Autonomic dysfunction may progress with reduction or lack of sweating, postural hypotension, deterioration of cardiac reflexes, change in the reaction of the skin to histamine, and abnormal methacholine test [10].

The aim of the present study was to discuss the application of general anesthesia to a patient with a diagnosis of AS who was planned to undergo Heller myotomy.

## 2. Case Report

A nine-year-old girl who had complaint of vomiting following eating and growth retardation after birth had been diagnosed with achalasia at the age of one. Endoscopic balloon dilation for achalasia had been performed for three times. The patient was one year old when she underwent the first endoscopic trial. No complications emerged during the endoscopic procedures. Allgrove syndrome was diagnosed in the patient due to adrenal insufficiency, developed nearly a year ago; consequently, hydrocortisone therapy was initiated.

The typical facial appearance of Allgrove syndrome including long philtrum, narrow upper lip, and fish mouth was observed during the preoperative assessment. At the same time retardation of growth and development, hyper-nasal speech, alacrima and adrenal insufficiency were observed. Her weight are only fifteen kilograms, hypernasal speech, alacrima, and adrenal insufficiency were observed. Oral hydrocortisone was also used due to adrenal insufficiency. Physical examination and interpretation of laboratory tests were defined normal (fasting blood glucose: 6,6 mmol/L, Na: 143 mEq/L, and K: 3,5 mEq/L). Postural tests were performed to assess autonomic dysfunction. We observed a decrease of approximately 15 mmHg in systolic artery pressure and an increase about 10 mm Hg in diastolic artery pressure. We concluded that autonomic dysfunction did not exist. The doses of steroids to be applied during the postoperative and preoperative periods were determined in the pediatric clinic. The patient, assessed in ASA risk group III, was given no premedication before anesthesia. Prednisolone 18 mg was administered intravenously on the night before surgery. The preoperative blood glucose level was recorded as 11 mmol/L.

The ECG, noninvasive blood pressure and SpO_2_ of the patient who was transferred onto the operating table, were monitored; infusion fluid which includes NaCl (4,5 g/l) and glucose (25 g/l) was started at the speed of 125 mL/h, because preoperative fluid deficit was given before. The patient was intubated with a 4 mm ID cuffed endotracheal tube after the anesthesia was induced by 75 mg of thiopental sodium, 1 mg of vecuronium, and 20 *μ*cg fentanyl. For protective purposes topical polymyxin ophthalmic ointment was used an the eyes before they were covered. Anesthesia was maintained with 2% sevoflurane in concentration of 50% O_2_ and 50% N_2_O. Mechanical ventilation was continued to be ETCO_2_ 30–40 mmHg. Prednisolone 25 mg was infused preoperatively. Monitoring of blood glucose was performed hourly.

In perioperative and postoperative period, we did not observe a condition that requires the intervention at HR, SAP and SpO_2_ (Table 1).

In intraoperative period, blood glucose was measured 12 mmol/L was at the first hour and 18 mmol/L at the second hour. Then, vascular access was opened and 0.9% NaCl infusion was started at 100 mL/h. 50 U regular insulin in 50 mL of 0.9% NaCl solution was prepared and it started infusion at 1,5 mL/h. Fluid infusion rate was reduced from 125 mL/h to 50 mL/h, and 15 mEq KCl was added. The operation is completed at 135 minutes in other words 15 minutes after the start of insulin infusion. Blood glucose was measured 13 mmol/L at postoperative 30th minute and insulin infusion rate was reduced to 0.7 mL/h. Because blood glucose decreased to 10 at postoperative first hour, insulin infusion was stopped.

Tramadol 30 mg is administrated by intravenous route as postoperative analgesic. There were no problems at extubation nor recovery from anesthesia. Consciousness, HR, SAP, SpO_2_, and respiration rate and pattern were normal. Blood glucose, Na, and K levels were measured 200, 148, and 3.2, respectively, at postoperative twelve hour, respectively.

The patient did not have any preoperative or postoperative anesthetic problems except a rise in the blood glucose level. The patient was discharged from the hospital on postoperative day 5.

## 3. Discussion

Allgrove syndrome is a multisystem disease associated with ACTH-resistant adrenal insufficiency, alacrima, achalasia, and autonomic dysfunction. Approach to endoscopic balloon dilatation or indication for surgical treatment is required in patients with Allgrove syndrome due to pre-existing achalasia. Undefined or inadequately treated adrenal insufficiency at the preoperative period may cause preoperative adrenal crisis consisting of shock, hyponatremia, hyperkalemia, or hypoglycemia. Cushing syndrome may develop also in these patients with the excessive use of glucocorticoids [11]. They may cause life-threatening conditions such as severe hypoglycemia which may result in an incognizable coma [12, 13]. Therefore, the stress-dose steroids should be used cautiously during the application of anesthesia [11]. Glycemic control should be closely monitored due to the contribution of the administered steroid doses to hyperglycemia induced by anesthesia and surgery. If necessary, insulin involvement should be applied. Serum electrolytes did not change significantly. Blood glucose had risen which requires insulin therapy in the patient.

Autonomic, motor, and sensory neuropathies, cerebellar ataxia, progressive spastic tetraparesis, increased nerve conduction time, and development of mental retardation are quite frequent in these patients. Regurgitation- and aspiration-associated gastroparesis may develop intraoperatively and postoperatively besides autonomic-neuropathy-associated heart rate variations, orthostatic hypotension, and differences in response to the deep respiration and Valsalva maneuvers. Since the influences of the hemodynamic state may emerge especially during position changes, they should be performed slowly, delicately, and cautiously [13, 14]. We thought originate from which autonomic dysfunction has not yet begun in the patients the reason why hemodynamic impairment have not saw at perioperative period.

Swallowing difficulty and vomiting associated with achalasia are accompanied by regurgitation, aspiration, and associated conditions as well. Pneumonia and respiratory failure associated with regurgitation and aspiration may increase [15, 16]. Consequently, the risk for aspiration in patients with Allgrove syndrome should be observed especially during the induction phase and rapid sequence intubation should be performed. Cuffed endotracheal tubes should be preferred to prevent another aspiration in these patients with high aspiration risk.

Another noteworthy point in our 9-year-old subject was the application of intubation using an endotracheal tube whose inner diameter was 4 mm. Although diameter of the tube was calculated as small according to patient's age, it provided effective breathing. This situation was thought associated with the present retardation in growth and development. We believe that loss of weight associated with achalasia and adrenal insufficiency in patients especially with late diagnosis and inadequate therapy may cause retardation in growth and development.

Disappearance of protective corneal reflexes, reduced tear production, and the loss of pain sensation during general anesthesia application result in the development of ocular complications. Protection of eyes under general anesthesia comes into question when the ocular influences such as alacrima-associated keratopathy and ulceration are also combined with the above situation in patients with AS [7, 9], [17]. In our case, because of the taken measures to protect the eyes, there was no symptoms associated with the patient's visual at postoperative period.

AS specializes application of anesthesia owing to its present components. We should feel alerted for a possible diagnosis of AS in patients with achalasia. The presence of adrenal insufficiency, appropriate therapy, and regulation of additional steroid doses should be assessed as well as the evaluation of achalasia-associated respiratory problems, retardation of growth and development, and autonomic and ophthalmic influences. We suggest caution for the application of steroids, because the risk of aspiration during anesthesia. We believe that a safe anesthesia can be achieved with very careful preoperative examination, application of steroids, control of glycemia, consideration of risk aspiration, and protection of the eyes.

## Figures and Tables

**Table 1:** Hemodynamic changes.

	HR (beat/minute)	SAP (mmHg)	SpO_2_ (%)
Preinduction	90	120	97
Induction	120	100	100
Postinduction	110	110	99
10 minutes	100	95	99
20 minutes	95	95	99
30 minutes	80	90	99
40 minutes	80	90	99
50 minutes	85	90	99
60 minutes	85	90	99
70 minutes	85	95	99
80 minutes	80	95	99
90 minutes	85	100	99
100 minutes	85	100	98
110 minutes	85	95	99
120 minutes	85	95	99
130 minutes	95	125	99
Recovery	120	130	98
Postop 10 Minutes	115	105	98
Postop 20 Minutes	100	95	97
Postop 30 minutes	100	100	98
